# Seasonal and Spatially Distributed Viral Metagenomes from Comau Fjord (42°S), Patagonia

**DOI:** 10.1128/mra.00082-23

**Published:** 2023-03-22

**Authors:** Eduardo Castro-Nallar, Valentín Berríos-Farías, Beatriz Díez, Sergio Guajardo-Leiva

**Affiliations:** a Departamento de Microbiología, Facultad de Ciencias de la Salud, Universidad de Talca, Talca, Chile; b Centro de Ecología Integrativa, Universidad de Talca, Talca, Chile; c Department of Molecular Genetics and Microbiology, Pontificia Universidad Católica de Chile, Santiago, Chile; d Center for Climate and Resilience Research (CR)2, Santiago, Chile; e Millennium Institute Center for Genome Regulation (CGR), Santiago, Chile; DOE Joint Genome Institute

## Abstract

Viruses are key players in marine environments, affecting food webs and biogeochemical cycles. We present 48 viral metagenomes and 5,656 viral operational taxonomic units (vOTUs) from Comau Fjord, Patagonia (42°S), to understand viral-mediated processes in coastal and estuarine waters. These data represent a spatial (35-km transect, two depths) and seasonal (winter and fall) data set.

## ANNOUNCEMENT

The ocean harbors a vast viral diversity that is crucial to its functioning (1, 2). However, our understanding of viruses in the sea is mainly based on global surveys of the open ocean (3–6), neglecting fjords and estuaries despite their ecological importance and the ecosystem services that they provide (7). Comau Fjord, Patagonia, is a semiclosed basin whose waters harbor open-cage aquaculture centers and are used for commercial and passenger transport routes (8). Here, we present a data set to uncover viral diversity and the ecological functions of viruses in a model fjord impacted by intensive anthropogenic activity.

We collected seawater (20 L per sample) in two seasons over 2 years at 5 and 20 m depth, from the mouth to the end of the fjord (Table 1). Macroscopic organisms, eukaryotes, particle-attached prokaryotes, and free-living prokaryotes were removed using sequential filtration through a 50-μm nylon mesh (Sefar), 3-μm polycarbonate filters (Merck, Millipore), and 0.22-μm polyethersulfone (PES) filters (Sterivex; Merck, Millipore). Viruses were concentrated using the FeCl_3_ flocculation method (9), and DNA was obtained using the PureLink viral RNA/DNA minikit (Invitrogen). Sequencing libraries (2 × 150 bp) were constructed using Illumina TruSeq DNA kits and sequenced in an Illumina NovaSeq 6000 sequencer, obtaining 2.3 to 11.2 million reads (average, 4.8 million) per library (Table 1).

**TABLE 1:** Summary information of the samples sequenced in this viral metagenome shotgun project

BioSample accession no.	Sample	File	No. of raw reads	No. of QC reads^*a*^	No. of mapped vOTU reads	Latitude	Longitude	Collection date	Yr	Season	Line	Site	Depth (m)	Coassembly cluster
SAMN19609973	Aug_2018_L1S1D05	VIR3A	4,348,632	3,856,017	1,015,972	−42.47733	−72.43112	26 August	2018	Winter	L1	S1	5	Cluster 1
SAMN19609974	Aug_2018_L1S1D20	VIR3B	5,260,185	4,228,661	521,352	−42.47733	−72.43112	26 August	2018	Winter	L1	S1	20	Cluster 1
SAMN19609975	Aug_2018_L1S2D05	VIR3C	2,735,214	2,328,294	297,584	−42.47294	−72.41806	26 August	2018	Winter	L1	S2	5	Cluster 1
SAMN19609976	Aug_2018_L1S2D20	VIR3D	4,875,373	3,808,166	499,268	−42.47294	−72.41806	26 August	2018	Winter	L1	S2	20	Cluster 1
SAMN19609977	Aug_2018_L1S3D05	VIR3E	3,207,663	1,971,431	527,594	−42.4731	−72.40573	26 August	2018	Winter	L1	S3	5	Cluster 1
SAMN19609978	Aug_2018_L1S3D20	VIR3F	4,058,399	2,919,219	301,956	−42.4731	−72.40573	26 August	2018	Winter	L1	S3	20	Cluster 1
SAMN19609979	Aug_2018_L3S1D05	VIR3G	10,844,995	7,655,435	1,175,388	−42.38636	−72.45599	22 August	2018	Winter	L3	S1	5	Cluster 1
SAMN19609980	Aug_2018_L3S1D20	VIR3H	11,326,118	10,281,661	2,388,994	−42.38636	−72.45599	22 August	2018	Winter	L3	S1	20	Cluster 1
SAMN19609981	Aug_2018_L3S2D05	VIR3I	3,813,802	3,040,939	308,182	−42.38078	−72.43892	22 August	2018	Winter	L3	S2	5	Cluster 1
SAMN19609982	Aug_2018_L3S2D20	VIR3J	5,328,156	4,496,909	1,630,110	−42.38078	−72.43892	22 August	2018	Winter	L3	S2	20	Cluster 1
SAMN19609983	Aug_2018_L3S3D05	VIR3K	4,043,699	3,116,747	746,122	−42.3747	−72.43074	22 August	2018	Winter	L3	S3	5	Cluster 1
SAMN19609984	Aug_2018_L3S3D20	VIR3L	6,086,675	5,056,556	1,691,999	−42.3747	−72.43074	22 August	2018	Winter	L3	S3	20	Cluster 1
SAMN19609985	Aug_2018_L4S1D05	VIR3M	3,027,632	1,994,406	248,841	−42.3066	−72.51493	29 August	2018	Winter	L4	S1	5	Cluster 1
SAMN19609986	Aug_2018_L4S1D20	VIR3N	4,272,438	3,578,428	642,369	−42.3066	−72.51493	29 August	2018	Winter	L4	S1	20	Cluster 1
SAMN19609987	Aug_2018_L4S2D05	VIR3O	3,928,225	3,370,167	369,651	−42.29935	−72.493	29 August	2018	Winter	L4	S2	5	Cluster 1
SAMN19609988	Aug_2018_L4S2D20	VIR3P	3,731,478	3,009,925	266,471	−42.29935	−72.493	29 August	2018	Winter	L4	S2	20	Cluster 1
SAMN19609989	Aug_2018_L4S3D05	VIR3Q	5,604,749	4,303,650	1,045,999	−42.26195	−72.43245	29 August	2018	Winter	L4	S3	5	Cluster 1
SAMN19609990	Aug_2018_L4S3D20	VIR3R	2,508,871	1,891,383	300,672	−42.26195	−72.43245	29 August	2018	Winter	L4	S3	20	Cluster 1
SAMN19609991	Aug_2018_L5S1D05	VIR3S	3,404,091	2,273,053	354,224	−42.20583	−72.54705	24 August	2018	Winter	L5	S1	5	Cluster 1
SAMN19609992	Aug_2018_L5S1D20	VIR3T	3,221,110	2,464,822	330,960	−42.20583	−72.54705	24 August	2018	Winter	L5	S1	20	Cluster 1
SAMN19609993	Aug_2018_L5S2D05	VIR3U	5,187,393	4,234,801	566,014	−42.20202	−72.49854	24 August	2018	Winter	L5	S2	5	Cluster 1
SAMN19609994	Aug_2018_L5S2D20	VIR3V	4,099,798	3,208,406	531,316	−42.20202	−72.49854	24 August	2018	Winter	L5	S2	20	Cluster 1
SAMN19609995	Aug_2018_L5S3D05	VIR3W	2,296,459	1,588,091	363,541	−42.17998	−72.45506	24 August	2018	Winter	L5	S3	5	Cluster 1
SAMN19609996	Aug_2018_L5S3D20	VIR3X	5,843,271	5,224,723	574,088	−42.17998	−72.45506	24 August	2018	Winter	L5	S3	20	Cluster 1
SAMN19609997	Mar_2019_L1S1D05	VIR4A	3,638,005	2,220,302	1,185,559	−42.47733	−72.43112	28 March	2019	Fall	L1	S1	5	Cluster 2
SAMN19609998	Mar_2019_L1S1D20	VIR4B	4,012,057	3,203,443	1,588,459	−42.47733	−72.43112	28 March	2019	Fall	L1	S1	20	Cluster 2
SAMN19609999	Mar_2019_L1S2D05	VIR4C	5,894,549	4,776,618	2,333,553	−42.47294	−72.41806	28 March	2019	Fall	L1	S2	5	Cluster 2
SAMN19610000	Mar_2019_L1S2D20	VIR4D	5,686,527	3,783,847	2,280,283	−42.47294	−72.41806	28 March	2019	Fall	L1	S2	20	Cluster 2
SAMN19610001	Mar_2019_L1S3D05	VIR4E	3,961,555	2,470,188	1,363,939	−42.4731	−72.40573	28 March	2019	Fall	L1	S3	5	Cluster 2
SAMN19610002	Mar_2019_L1S3D20	VIR4F	3,237,181	2,135,334	1,163,923	−42.4731	−72.40573	28 March	2019	Fall	L1	S3	20	Cluster 2
SAMN19610003	Mar_2019_L3S1D05	VIR4G	6,012,860	3,620,809	1,892,292	−42.38636	−72.45599	2 April	2019	Fall	L3	S1	5	Cluster 2
SAMN19610004	Mar_2019_L3S1D20	VIR4H	7,921,010	5,252,696	3,214,423	−42.38636	−72.45599	2 April	2019	Fall	L3	S1	20	Cluster 2
SAMN19610005	Mar_2019_L3S2D05	VIR4I	5,462,094	3,639,041	2,105,364	−42.38078	−72.43892	2 April	2019	Fall	L3	S2	5	Cluster 2
SAMN19610006	Mar_2019_L3S2D20	VIR4J	4,109,021	2,738,155	1,609,943	−42.38078	−72.43892	2 April	2019	Fall	L3	S2	20	Cluster 2
SAMN19610007	Mar_2019_L3S3D05	VIR4K	5,182,456	3,472,414	1,773,417	−42.3747	−72.43074	2 April	2019	Fall	L3	S3	5	Cluster 2
SAMN19610008	Mar_2019_L3S3D20	VIR4L	6,762,123	5,565,745	3,916,908	−42.3747	−72.43074	31 March	2019	Fall	L3	S3	20	Cluster 2
SAMN19610009	Mar_2019_L4S1D05	VIR4M	5,151,726	3,110,351	1,313,924	−42.3066	−72.51493	31 March	2019	Fall	L4	S1	5	Cluster 2
SAMN19610010	Mar_2019_L4S1D20	VIR4N	3,423,016	2,317,372	1,250,433	−42.3066	−72.51493	31 March	2019	Fall	L4	S1	20	Cluster 2
SAMN19610011	Mar_2019_L4S2D05	VIR4O	4,519,742	2,873,192	1,335,014	−42.29935	−72.493	31 March	2019	Fall	L4	S2	5	Cluster 2
SAMN19610012	Mar_2019_L4S2D20	VIR4P	3,448,897	2,107,265	1,201,592	−42.29935	−72.493	31 March	2019	Fall	L4	S2	20	Cluster 2
SAMN19610013	Mar_2019_L4S3D05	VIR4Q	5,859,239	3,721,781	1,742,866	−42.26195	−72.43245	31 March	2019	Fall	L4	S3	5	Cluster 2
SAMN19610014	Mar_2019_L4S3D20	VIR4R	7,434,900	6,870,357	4,118,645	−42.26195	−72.43245	26 March	2019	Fall	L4	S3	20	Cluster 2
SAMN19610015	Mar_2019_L5S1D05	VIR4S	4,949,977	3,105,639	1,583,847	−42.20583	−72.54705	26 March	2019	Fall	L5	S1	5	Cluster 2
SAMN19610016	Mar_2019_L5S1D20	VIR4T	5,803,592	4,736,743	3,013,534	−42.20583	−72.54705	26 March	2019	Fall	L5	S1	20	Cluster 2
SAMN19610017	Mar_2019_L5S2D05	VIR4U	5,148,745	3,325,444	1,791,672	−42.20202	−72.49854	26 March	2019	Fall	L5	S2	5	Cluster 2
SAMN19610018	Mar_2019_L5S2D20	VIR4V	4,402,817	3,881,807	2,178,116	−42.20202	−72.49854	26 March	2019	Fall	L5	S2	20	Cluster 2
SAMN19610019	Mar_2019_L5S3D05	VIR4W	3,735,551	2,800,001	1,738,375	−42.10724	−72.27295	26 March	2019	Fall	L5	S3	5	Cluster 2
SAMN19610020	Mar_2019_L5S3D20	VIR4X	3,381,167	2,184,737	1,141,622	−42.10724	−72.27295	26 March	2019	Fall	L5	S3	20	Cluster 2

a QC, quality checked.

We used default software parameters unless otherwise stated. We removed adapters (–detect_adapter_for_pe) and carried out filtering and trimming (-q 30 -l 100) using fastp v0.23.2 (10). We first estimated distances using MASH v2.3 (11) and measured the degree of clustering using the Hopkins statistics (12) (>0.75). We applied the hclust function (R base) for hierarchical clustering and evaluated the clustering quality using silhouette analysis (13), which resulted in two clusters (Table 1). *De novo* metagenome coassembly was carried out on each cluster as implemented using MEGAHIT v1.2.9 (–min-contig-len 1500) (14). Contigs (length, >10 kb) were dereplicated using cd-hit v4.8.1 (15) (cd-hit-est) at 95% identity and 80% coverage. The resulting nonredundant contigs were analyzed using geNomad v1.1.0 (–min-score 0.7) (16) to identify and assign taxonomy to putative viral sequences and CheckV v1.0.1 (17) to assess their quality and completeness. Viral sequences with a CheckV quality of “not determined” were removed from the viral operational taxonomic unit (vOTU) set. To obtain the read abundances per vOTU, we mapped the reads from each sample against the vOTUs using Bowtie2 v2.3.5.1 (18) (-end-to-end -sensitive).

**FIG 1:**
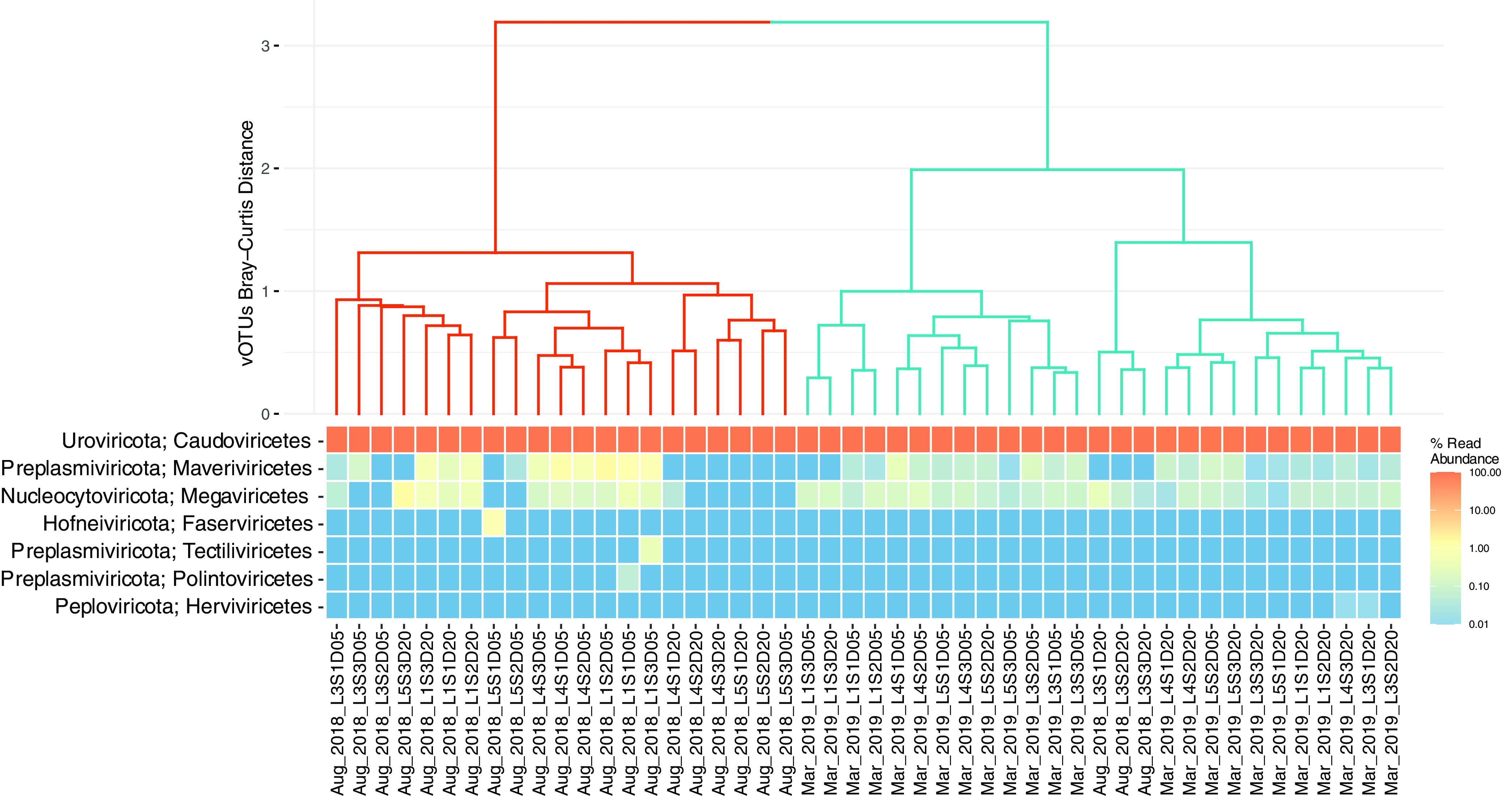
Bray-Curtis dissimilarity cluster analysis and viral taxonomic composition. The dendrogram was obtained by hierarchical clustering of the samples based on their Bray-Curtis dissimilarity calculated from the vOTU matrix. The heat map represents the taxonomic composition of each sample obtained by mapping reads to each vOTU, agglomerated at the class level.

The 5,656 vOTUs presented here had different quality indices, as determined using CheckV: 159 vOTUs were complete, 263 vOTUs were of high quality, 782 vOTUs were of medium quality, and 4,452 vOTUs were of low quality. Only 464 vOTUs were classified beyond the class level, while most sequences (98%) were assigned to the order *Caudoviricetes* (Fig. 1). Additionally, the orders *Maveriviricetes* (0.2%) and *Megaviricetes* (0.14%) were detected across most of the samples (Fig. 1). Finally, the orders *Tectiliviricetes*, *Polintoviricetes*, and *Herviviricetes* were seen in some specific samples (Fig. 1). Clustering based on the vOTU composition suggested the existence of seasonal patterns—winter (red lines in Fig. 1) and fall (blue lines) clusters—in the species turnover of viral communities.

### Data availability.

This whole viral metagenome shotgun project has been deposited at GenBank under BioProject accession no. PRJNA734712. See Table 1 for the details of each sequenced sample. The version described in this paper is the first version. The vOTU data can be found at Figshare (https://doi.org/10.6084/m9.figshare.21989717).
